# Temperature or competition: Which has more influence on Mediterranean ant communities?

**DOI:** 10.1371/journal.pone.0267547

**Published:** 2022-04-29

**Authors:** Daniel Sánchez-García, Xim Cerdá, Elena Angulo

**Affiliations:** 1 Estación Biológica de Doñana, CSIC, Sevilla, Spain; 2 Museum and Institute of Zoology, Polish Academy of Science, Warszawa, Poland; 3 Laboratoire Écologie, Systématique, Évolution, Université Paris-Saclay, Orsay Cedex, France; Southeastern Louisiana University, UNITED STATES

## Abstract

Temperature and competition are two of the main factors determining ant community assemblages. Temperature may allow species to forage more or less efficiently throughout the day (in accordance with the maximum activity temperature of each species). Competition can be observed and quantified from species replacements occurring during resource exploitation. We studied the interspecific competitive interactions of ant communities from the Doñana Biological Reserve (southern Spain). Ants were sampled from pitfall traps and baits in three habitats with contrasted vegetation physiognomy (savin forest, pine forest, and dry scrubland). We measured the temperature during the competitive interactions between species and created a thermal competition index (TCI) to assess the relative contribution of temperature and numerical dominance to the competitive outcomes. Temperature had unequal effects on ant activity in each type of habitat, and modulated competitive interactions. The TCI showed that a species’ success during pair interactions (replacements at baits) was driven by the proportion of workers between the two competing species and by the species-specific effect of temperature (how advantageous the temperature change is for each species during bait replacement). During competitive interactions, the effect of temperature (higher values of TCI) and numeric supremacy (higher worker proportion) gave higher success probabilities. Interspecific competitive relationships in these Mediterranean ant communities are habitat dependent and greatly influenced by temperature.

## Introduction

Ants are one of the most abundant, successful and dominant invertebrate taxa in terrestrial ecosystems [1]. They are ecosystem engineers, capable of modifying their surrounding environment [2, 3]. Their eusocial lifestyle serves as a buffer against predation and environmental stress, and thus, interspecific competition has been long considered the widespread mechanism structuring ant communities [4, 5] (but see [6, 7]).

Early research on ant competition grouped species into transitive hierarchies, by which the superior behavioral competitor excludes (or limits and reduces) the subordinate species. Dominant ant species can monopolize space and food resources, influencing behavior and the abundance of co-occurring species [4, 8–13]. Later, other factors such as daily and seasonal temperature fluctuations were shown to disrupt transitive hierarchies allowing subordinate species to have greater ecological dominance [14]. Recently, dominance hierarchies in ant communities have been questioned [15], with a proposal to consider ant communities as networks of interacting species rather than linear hierarchies ranking from most to least dominant species. There is increasing evidence that there is no single monolithic process governing ant community organization, something that was already stated almost 40 years ago [9] when competition was proposed as just one of the many important factors or mechanisms affecting the assemblage structure of ants.

It is known that Mediterranean ecosystems have a wide range of temperature fluctuations [16] and these fluctuations may have an important effect on ectotherm animals, whose body temperature regulation depends on external sources (butterflies: [17, 18]; damselflies: [19]; bumble bees: [20]; spiders: [21–23]; herptiles: [24]). In the case of ants, many species have been shown to shift their foraging activity rhythms following daily or seasonal temperature changes to match their thermal preferences [25–29]. In accordance with their thermal preferences, ant species can be classified as heat-tolerant or heat-intolerant species if they mainly forage during the hottest hours of the day or when temperatures are much lower, respectively [30]. Mediterranean ant communities exhibit a trade-off between dominance and thermal tolerance: dominant species normally have lower maximum activity temperature values as they are heat-intolerant species, while subordinate species usually behave as heat-tolerant species, avoiding competition from dominants by overlapping their activity periods with them [30, 31].

This study aims to analyze the factors that drive Mediterranean ant assemblages in a relatively small spatial scale as is the Doñana Biological Reserve (6,794 ha within the Doñana National Park, SW Spain). Since habitat structure determines local temperature variability and the thermal environment [32]. We first compare the structure of the ant community in three different habitats, by studying the spatial and temporal co-occurrence patterns of their ant species during daylight hours (when the greatest temperature variation occurs). Second, we test whether the success of the interactions depends more on the thermal environment or on the life history traits relating to dominance. To do so, we implemented a new thermal competitive index. This index takes into account not only the change in temperature during interspecific interactions, but also how far each species is from its optimal foraging temperature when the interaction occurs. We hypothesize that dominance is mediated by temperature variations, which in turn are modulated by the local habitat structure. Specifically, we make the following predictions:

- Habitats with higher vegetation cover should contain a low range of thermal niches: vegetation cover buffers temperature variability. Thus, we expect an increased importance of competition in these habitats, and thus a segregation of spatial and temporal co-occurrence patterns are expected in these habitats.
- By contrast, open habitats will have a wider range of thermal niches that are expected to sustain different species that are active at different times of the day. Thus, competition will be less important because of the high thermal niche availability, and therefore, patterns of temporal niche overlap and spatial co-occurrence are expected to be random.

## Material and methods

### Study site

**Fig 1:**
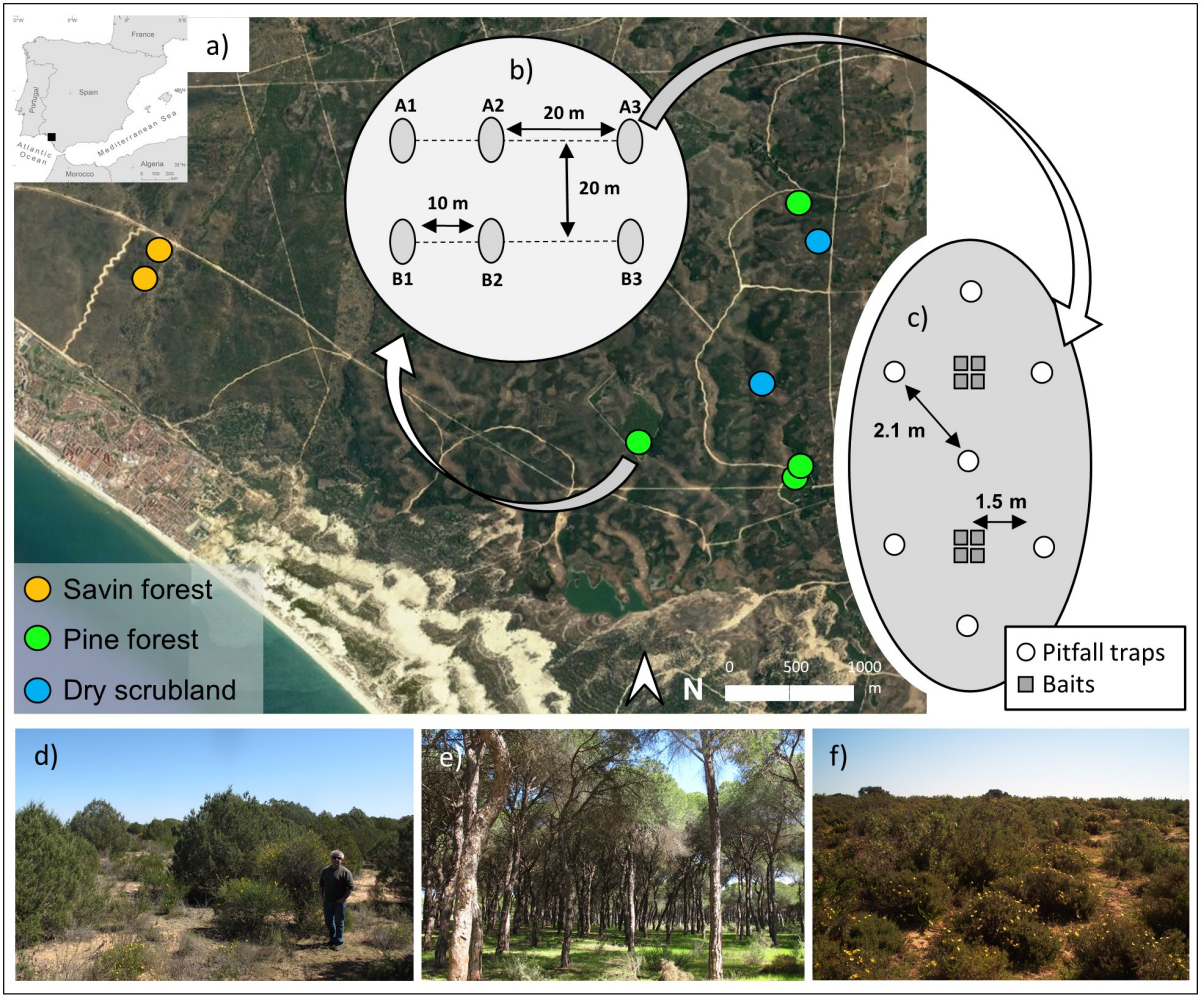
Study area and sampling design: (a) aerial view of the study area (Doñana Biological Reserve, SW Spain) showing sampling plot site for each habitat type; (b) schema of the two sampling transects (A and B), each transect consists of three sampling points; (c) each sampling point has 7 pitfall traps (white circles) and 8 baits (grey squares); d) savin juniper forest; e) pine forest; f) dry scrubland. Aerial view from digital aerial orthophotograph of the Spanish National Orthophoto Program (PNOA) (September 2019) under a CC BY 4.0 License (https://www.ign.es/wms-inspire/pnoa-ma? and free download through the CNIG’s download centre).

The field study was conducted in the Doñana Biological Reserve (37°1’N, 6°33’W; Doñana National Park, SW Spain). Doñana has a typical subhumid Mediterranean climate. The influence of the Atlantic Ocean softens temperatures, the annual average is 17°C, and the monthly averages oscillate between 9.9°C (January) and 24.7°C (July). The mean annual precipitation is 575 mm, most falling in winter [33]. Three different habitats have been studied: savin juniper forest (*Juniperus phoenicea*), pine forest (*Pinus pinea*) and a dry scrubland (Fig 1). Each habitat offers ants different environmental conditions. Pine forest and savin juniper forest present the lowest temperature fluctuations or the largest shadows, which ants can use to forage, while dry scrubland plots have more extreme temperature conditions. The sampling was conducted in summer, July and August of 2001. Two plots were established for the dry scrubland and the savin forest, and four plots were established in four different pine tree forests (Fig 1a). Permit for field work in Doñana National Park was authorized by Doñana’s Coordination of Research Commission (project reference 02/2001).

### Ant sampling

To sample ant presence, abundance and activity in each plot, we placed two transects distanced 20m from each other (Fig 1b). We placed three groups of pitfall traps and baits distanced 10m and 20m from each other (Fig 1b) in each transect. Each group consisted of seven pitfall traps that were spaced 1.5m from each other forming two diamonds and eight baits divided in two groups each inside one diamond (Fig 1c). This diamond formation facilitates a better detection of pitfalls in heterogeneous habitats where it is relatively difficult to do a grid.

Pitfall traps were 6 cm in diameter and 7 cm deep plastic vials partially filled with water and soap. They were placed on the ground for 24 h. The content of pitfall traps was preserved in 70% ethanol and analyzed in the laboratory to species level.

Ant foraging activity was measured at baits during the course of one summer day (July-August) in each habitat using the twelve groups of four baits of each study plot (but only in two of the four pine forest plots) (Fig 1b and 1c). Baits were small plastic cards with four different large food rewards with different nutritional contributions (cheese and chorizo: both protein+lipid baits; biscuit: sugar+lipid bait; and water-diluted honey: sugar bait), refilled as much as necessary. The distance between the cards of each group was 25cm. Baits were installed at 07:00 a.m., and the first bait observation was conducted at 08:00 a.m. (hours according to official time = solar time+2). Once an hour from dawn until it was completely dark (08:00 a.m. until 23:00 p.m.), the number of workers of each ant species feeding at each bait was recorded by one or two observers moving around baits of the plot. Ground surface temperatures near the baits were measured with HOBO’s Data-Loggers buried with a very thin sand layer (<10 mm). For each group of bait records, we assigned a temperature value from the sensor. Distances between the baits and temperature sensor were 1.5m.

### Measuring spatial co-occurrence and temporal niche overlap

The co-occurrence analysis tests whether the observed pattern of species occurrence in pitfall traps or baits differs from the pattern expected in the absence of the proposed mechanism, which in our case is interspecific competition on spatial and temporal levels. To analyze species co-occurrence, separate matrices for each sampled plot were created. Species presence/absence matrices were based on two different types of sampling: the pitfall trap groups or the baits of each plot. The presence/absence matrices were tested for non-random patterns of species spatial co-occurrence using the *cooc_null_model* function with the *sim9* algorithm and 10000 permutations (package EcoSimR, version 0.1.0, [34]). A C-score standardized effect size (SES) was obtained for each plot as the mean of five simulated SES. This represents the species community pattern: the community is segregated when SES is greater than 1.96; it is random when the score is between -1.96 and 1.96; and the community is aggregated when the SES is less than -1.96. Spatial segregation indicates that species spatially co-occur less frequently with each other than expected by chance (e.g. species are further apart than in a random distribution), and spatial aggregation indicates that species co-occur more frequently than expected by chance (e.g., when they share the microhabitat, nesting around the same plant species). Segregation is considered the result of competition [35–37], but see [38], while aggregation comes from the similar spatial requirements of species that are not dominant extirpators and can share resources.

The temporal niche overlap for each plot was calculated with bait occupation data to test whether foraging activity rhythms are or are not influenced by competition within habitats (i.e. whether species segregate or do not segregate their activity rhythms within habitats). Non-random patterns were tested similarly to the C-score spatial values with the *niche_null_model* function by the ra2 algorithm and 10000 permutations (package EcoSimR, version 0.1.0, [33]), resulting in a SES of the temporal niche overlap for each plot that is interpreted similarly to the SES of the C-score index. Temporal segregation indicates that species co-occur less frequently with each other than expected by chance (they have significantly different activity rhythms), and temporal aggregation indicates that species co-occur more frequently than expected by chance (they have very similar foraging activity patterns).

### Analyzing activity patterns in relation to the temperature

For studying the temperature niche species overlap, we built a quantitative unipartite network by using co-occurrence frequencies, defined as the total number of records in which two species are at the same bait at the same time, as a surrogate for interaction strength. Modularity was estimated by applying a simulated annealing approach [39] using the *netcarto* function (package rnetcarto, version 0.2.4, [40]). To evaluate whether species within the modules were organized in accordance with their optimal temperature, we used a multinomial logistic regression with module identity as response variable and the species optimal temperature as predictor variable. Multinomial logistic regressions allowed us to predict the probability of module membership based on the species’ optimal temperature for foraging.

Additionally, we analyzed the effect of temperature on bait occupation for the four most abundant species of each habitat. The bait occurrence was grouped for each species by 5°C intervals of temperature and expressed as the percentage of occupied baits over the total of baits registered at each temperature interval. The percentage of baits occupied by each species in each habitat was related to temperature using the *lm* function with Gaussian distribution from R [41]. We modeled linear and quadratic fits and obtained the equation fit for the most significant model using the adjusted R^2^ statistic.

### Predicting species’ competitive success: The thermal competition index

First, from all the bait occupation data of the study, we estimated the maximal activity temperature of foraging (MAT) for each species: MAT is the median of the temperatures recorded when the species is active at the baits. Second, from the differences between MAT and the temperature during interactions, we calculated the improvement rate (ImR), which takes into account the temperature change during each interaction. It estimates whether at the end of the interaction, this change results in a temperature closer, equal to or farther away from each species’ own MAT. The ImR can be calculated for both winner and loser species of the interaction, using their own MAT for each of them. The formula used depends on the MAT value referred to the temperature change that occurred, obtaining a positive, negative or 0 value. A positive ImR value means that the temperature approached the species’ own MAT at the end of the interaction, while a negative value means that the temperature was farther from the species’ own MAT at the end of the interaction. Conditional equations to calculate ImR are coded in an R function (S1 Appendix).

During bait provision, species replacements arrived when a species A at a given bait was replaced by another species B in the next hour. We considered that species A was the loser of the interaction while species B was the winner. The ImR is used to calculate the thermal competition index (TCI), which is used in our analysis, and describes if the change in temperature during the interaction is more or less beneficial for target species A than for species B with respect to their preferred foraging temperature (MAT). The TCI is positive when the change in temperature is more beneficial for A than for B; it is negative, when the change in temperature is less beneficial for target species A than for B; or it is zero, when both species have equally changed: they approached or were farther from their MAT.

The probability of success of each species was analyzed by performing a generalized linear model (GLM) using the *glm* function [41]. One GLM was performed for each species having sufficient data: *Aphaenogaster senilis*, *Cataglyphis* spp., *Crematogaster auberti*, C. *scutellaris*, *Plagiolepis pygmaea* and *Tapinoma* cf. *nigerrimum*. The success (yes or no) of target species A was the dependent variable, while the independent variables were: 1) worker proportion: the quotient between the worker numbers of target species A and the worker numbers of species B; 2) the identity of species B interacting with target species A; and 3) the thermal competition index (TCI), calculated by subtracting the improvement rate (ImR) of target species A from that of the species B with which it is interacting. The best model fit of the variables was tested using the *drop1* function and the F statistic [41].

Finally, we predicted the probability of success of the target species using the *predict* function [40] with three different combinations of the two independent variables: worker proportion (5:1, 1:1; 1:5), and TCI (+15, 0, -15). Not all the combinations of values expressed in the graph can be found in nature, so some modeled probabilities just represent a theoretical approach. We only used the most abundant species, which were the ones having at least 6 replacements; and for each species (i.e. target species) we ran a prediction. The prediction ranged from a minimum value of 0 when the species always lost, and a maximum value of 1 when the species always won.

## Results

### Species co-occurrence and temporal niche overlap

**Fig 2:**
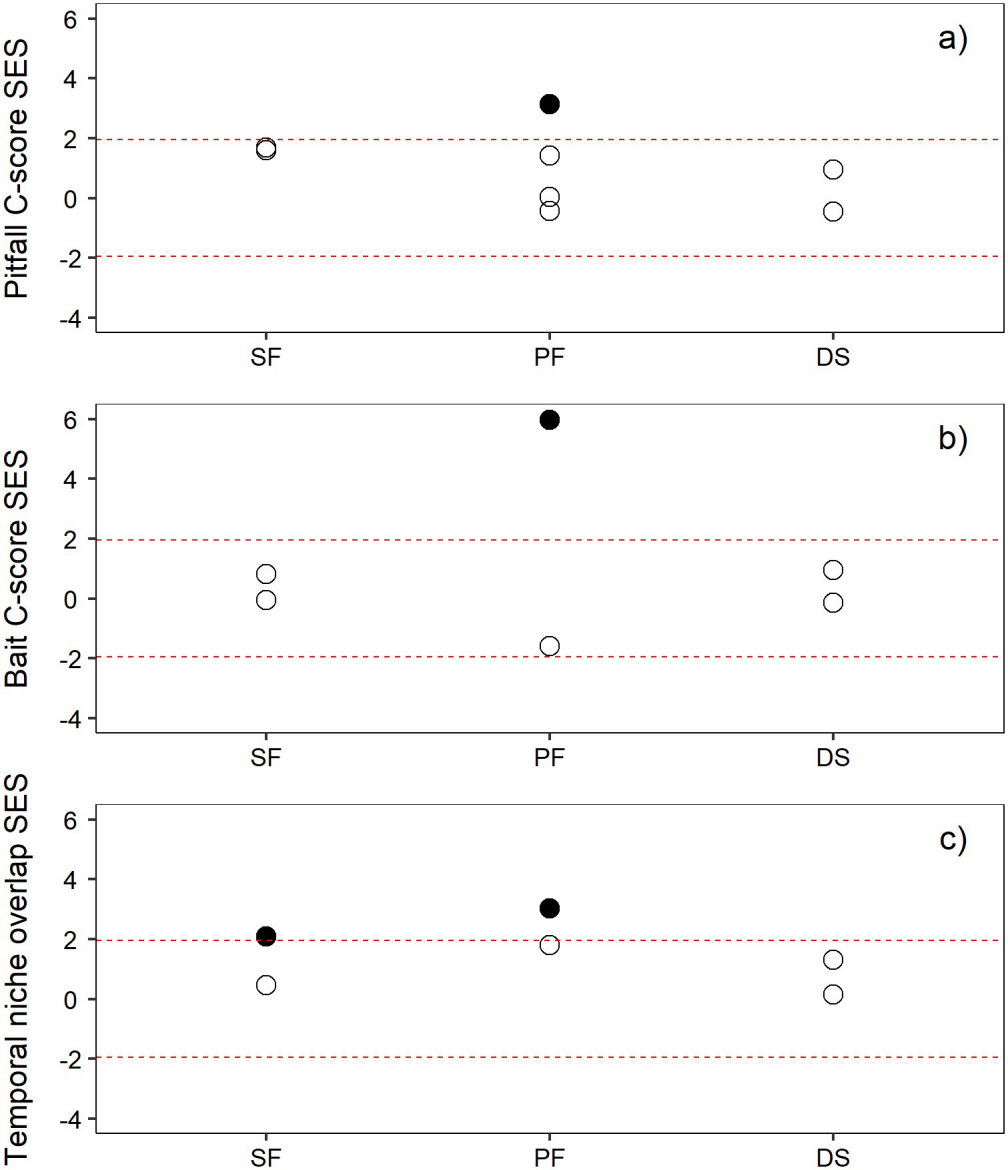
C-score standardized effect size (SES) values at each study habitat. (SF: Savin forest, PF: Pine forest, DS: Dry scrubland) from the C-score of (a) pitfall catches and (b) bait observations, and (c) temporal niche overlap. The dotted lines represent 1.96 standard deviation, the approximate level of statistical significance (p < 0.05). Thus, the larger standardized C-scores (filled circle), the less co-occurrence compared with a randomly assembled community, while values within the dotted lines (open circle) correspond to a random pattern of organization.

In analyzing the pitfall trap captures at each of the study sites (see S1 Table for the species list and abundances), we found that species co-occurred randomly (no pattern) at all sites, except for one of the pine forests, where the co-occurrence showed a segregation pattern (Fig 2a). Segregation in the pine forest indicated that the species co-occurred less frequently than expected by chance (significant high C-score), so in this plot, species had a separate spatial distribution, showing an exclusion pattern. When we analyzed the bait spatial data, we obtained the same results (Fig 2b). Species co-occurred randomly in all sites except for the same pine forest plot, where we found a segregation pattern again, indicating that there was competitive exclusion for resources between the different species.

When we analyzed the temporal niche overlap with the data from the species recorded in the baits at each hour, we found that one savin forest plot and one pine forest plot (but not the same one that presented the segregated pattern with the C-score index) showed a segregation pattern in the temporal foraging niche of their species (Fig 2c). We found that the temporal niche overlap was random (no pattern) for the rest of the plots and habitats.

### Temperature and bait exploitation

**Fig 3:**
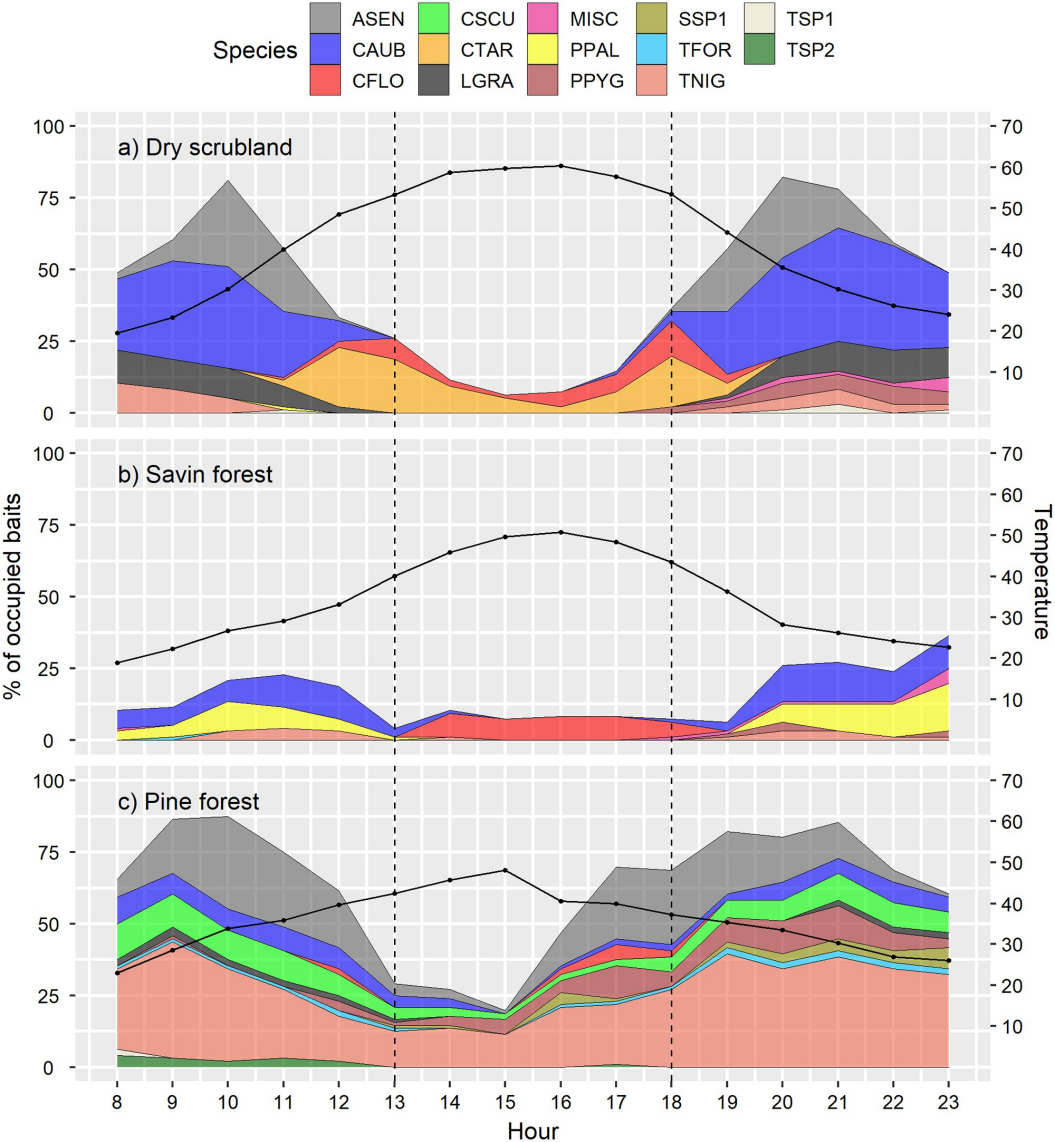
Bait occupation by hour in each habitat: (a) Dry scrubland, (b) Savin forest, and (c) Pine forest. Mean temperature for each habitat is shown with a black line and represented on the right axis. Dashed lines divide the hours in 3 periods: 8h to 13h and 18h to 23h –the coolest ones, and 13h to 18h –the warmest ones. Species abbreviations: ASEN (*Aphaenogaster senilis*), CAUB (*Crematogaster auberti*), CFLO (*Cataglyphis floricola*), CSCU (*Crematogaster scutellaris*), CTAR (*Cataglyphis tartessica*), LGRA (*Lasius grandis*), Miscellaneous (less abundant species), PPAL (*Pheidole pallidula*), PPYG (*Plagiolepis pygmaea*), SSP1 (*Solenopsis* sp.), TFOR (*Tetramorium forte*), TNIG (*Tapinoma* cf. *nigerrimum*), TSP1 (*Temnothorax sp*. 1) and TSP2 (*Temnothorax sp*. 2).

Foraging activity rhythms were measured to estimate how species are distributed throughout the day. We found the highest bait occupations for any studied habitat from 8h to 13h and 18h to 23h, while the lowest was from 13h to 18h (Fig 3). The pine forest had the highest bait occupation for 3 time periods (10h: 87.5%, 17h: 69.8% and 21h: 85.4%), followed closely by the dry scrubland (10h: 81.3%, 18h: 37.5% and 20h: 82.3%) and the lowest values were in the savin forest (11h: 22.9%, 14h: 10.4% and 23h: 36.5%).

Comparing bait occupation by temperature, we found the maximum occupation at 35°C in the dry scrubland and pine forest (87.5% and 81.4%, respectively), while in the savin forest, it was at 25°C (24.7%) (S1 Fig). Dry scrubland had the full range of temperatures registered (20–65°C), while the savin forest started at 20°C and ended at 55°C and pine forest started at 25°C and finished at 65°C. It is notable how the species and their occupation rates changed throughout the day depending on the ground temperature (S1 Fig).

**Fig 4:**
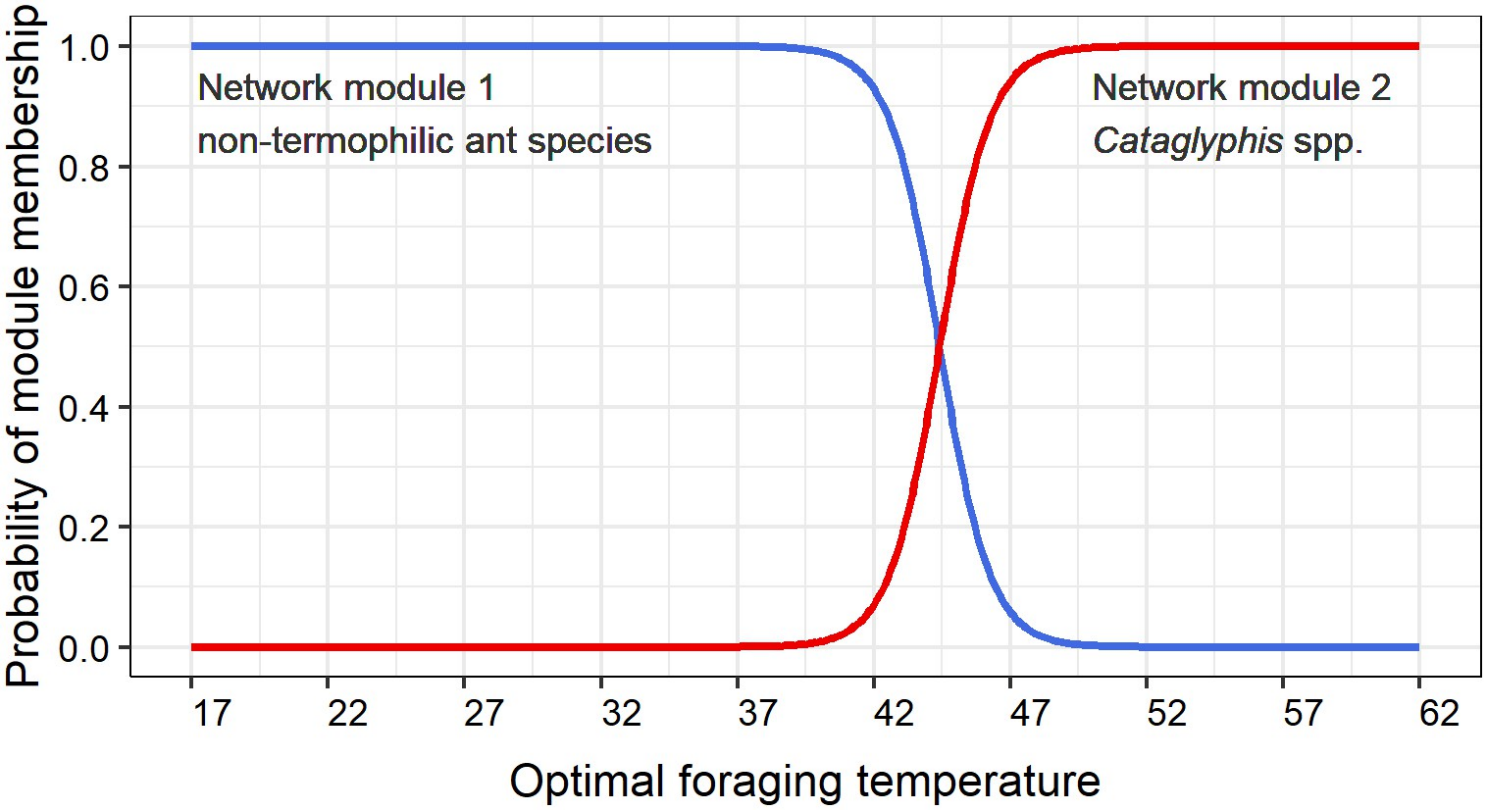
Probability of network module membership based on the species’ optimal foraging temperature.

Network modularity shows 2 groups of species more intensely connected. Network module 1 contains the 11 species with the lowest optimal foraging temperature (30°C on average) and its probability of module membership increases by decreasing the species’ optimal foraging temperature. Network module 2 contains the only two species of the thermophilic genus *Cataglyphis* recorded in our samplings, with the two highest optimal foraging temperatures (54.4°C on average) and its probability module membership increases by increasing the species’ optimal foraging temperature (Fig 4).

**Fig 5:**
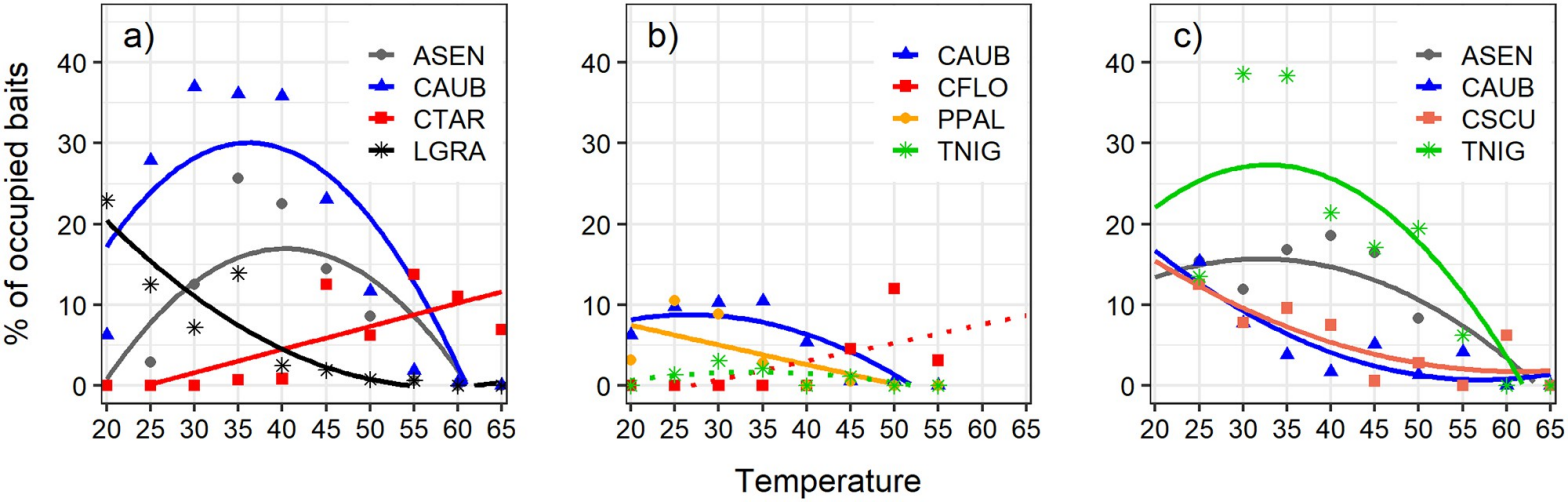
Linear or quadratic fit of the effect of temperature on bait occupation for the four most abundant species in each habitat: (a) Dry scrubland, (b) Savin forest and (c) Pine forest. Species abbreviations: ASEN (*Aphaenogaster senilis*), CFLO (*Cataglyphis floricola*), CTAR (*Cataglyphis tartessica*), CAUB (*Crematogaster auberti*), CSCU (*Crematogaster scutellaris*), LGRA (*Lasius grandis*), PPAL (*Pheidole pallidula*) and TNIG (*Tapinoma* cf. *nigerrimum*). Significant fits are shown by a solid line, not significant fits with a dotted line (S2 Appendix).

The thermal requirements of the four most abundant species in each habitat showed different patterns in their activity in the different habitats. The relationship between bait occupation and temperature in the dry scrubland followed a quadratic pattern for the three less thermotolerant species analyzed: *Crematogaster auberti* and *Aphaenogaster senilis* showed an increase of occupation until 36.4°C and 39.5°C respectively, followed by a decrease at higher temperatures (*C*. *auberti*, y = -34.235 + 3.554x − 0.049x^2^, R^2^ = 0.64, p = 0.012; *A*. *senilis*, y = -46.644 + 3.153x + -0.039x^2^, R^2^ = 0.56, p = 0.023) (Fig 5a) while *Lasius grandis* presented a continuous decrease in occupation with increasing temperatures (*L*. *grandis*, y = 47.655–1.642x + 0.014x^2^, R^2^ = 0.82, p = <0.001) (Fig 5a). Meanwhile, the relationship for the thermophilic *Cataglyphis tartessica* fit a positive linear pattern (y = -6.987 + 0.287x, R^2^ = 0.55, p = 0.009) (Fig 5a).

At the savin forest, *Crematogaster auberti* fits a quadratic pattern, showing a small increase in bait occupancy up to 26.8°C, followed by a decrease at higher temperatures (y = -0.908 + 0.728x − 0.014x^2^, R^2^ = 0.67, p = 0.026); for *Tapinoma* cf. *nigerrimum*, this relationship was not significant (y = -4.313 + 0.352x − 0.005x^2^, R^2^ = 0.20, p = 0.250) (Fig 5b). *Pheidole pallidula*, which is not a heat-tolerant species, showed a negative linear relationship between bait occupation and temperature (y = 12.334–0.243x, R^2^ = 0.42, p = 0.049) (Fig 5b). *Cataglyphis floricola*, a heat-tolerant species, increased its bait occupation with the increase of temperature but the relationship was not statistically significant (y = -6.062 + 0.227x, R^2^ = 0.34, p = 0.077) (Fig 5b).

At the pine tree forest, the four species followed a quadratic pattern. Two of them, *T*. cf. *nigerrimum* and *A*. *senilis*, showed an increase of occupation until 32.7°C and 31.8°C respectively, followed by a decrease at higher temperatures (*T*. cf. *nigerrimum*, y = -7.366 + 2.116x − 0.032x^2^, R^2^ = 0.58, p = 0.032; *A*. *senilis*, y = -0.489 + 1.011x − 0.016x^2^, R^2^ = 0.63, p = 0.021) (Fig 5c). Meanwhile, the other two species, *Crematogaster scutellaris* and *C*. *auberti*, showed a similar continuous decrease as the temperature increased (*C*. *scutellaris*, y = 31.904–0.984x + 0.008x^2^, R^2^ = 0.59, p = 0.030; *C*. *auberti*, y = 38.534–1.323x + 0.012x^2^, R^2^ = 0.69, p = 0.013) (Fig 5c).

### Replacement success: Affecting variables and prediction of success

For each species, we tested three variables that could affect its success in the interactions: the thermal competition index, worker proportion and the identity of the opponent species. Among the variables affecting the success of species replacement, the thermal competition index was statistically significant in five of the seven analyzed species (*A*. *senilis*, *Cataglyphis* spp., *C*. *auberti*, *C*. *scutellaris* and *L*. *grandis*) (Table 1). Worker proportion was significant for two of these species (*A*. *senilis* and *C*. *scutellaris*), while the identity of the opponent species was significant only for one species (*C*. *scutellaris*). None of these variables significantly affected the replacement success of *Plagiolepis pygmaea* and *T*. cf. *nigerrimum*.

**Table 1:** Number of bait observations (N) and effects of TCI (thermal competition index), relative ant proportion and interaction with, on the probability of having success during the interaction.

		TCI		Ant proportion		Interaction with	
Species	N	F	p	F	p	F	p
*Aphaenogaster senilis* (ASEN)	99	**18.67**	**<0.001**	**23.28**	**<0.001**	0.90	0.526
*Cataglyphis floricola* and *tartessica* (CATA)	38	**128.72**	**<0.001**	1.24	0.275	0.20	0.974
*Crematogaster auberti* (CAUB)	47	**4.33**	**0.044**	2.62	0.114	1.12	0.374
*Crematogaster scutellaris* (CSCU)	17	**2.92e+10**	**<0.001**	**1.76e+10**	**<0.001**	**4.37e+9**	**<0.001**
*Lasius grandis* (LGRA)	10	**4.36e+10**	**<0.001**	1.05	0.363	1.01	0.475
*Plagiolepis pygmaea* (PPYG)	22	1.13	0.308	0.19	0.670	0.89	0.895
*Tapinoma* cf. *nigerrimum* (TNIG)	30	0.42	0.534	1.99	0.173	1.47	0.235

‘Ant proportion’ refers to the coefficient between the number of individuals of the target species and the number of individuals of the opponent species. ‘Interaction with’ refers to the opponent species involved in the interaction. Significant variables are marked in bold.

**Fig 6:**
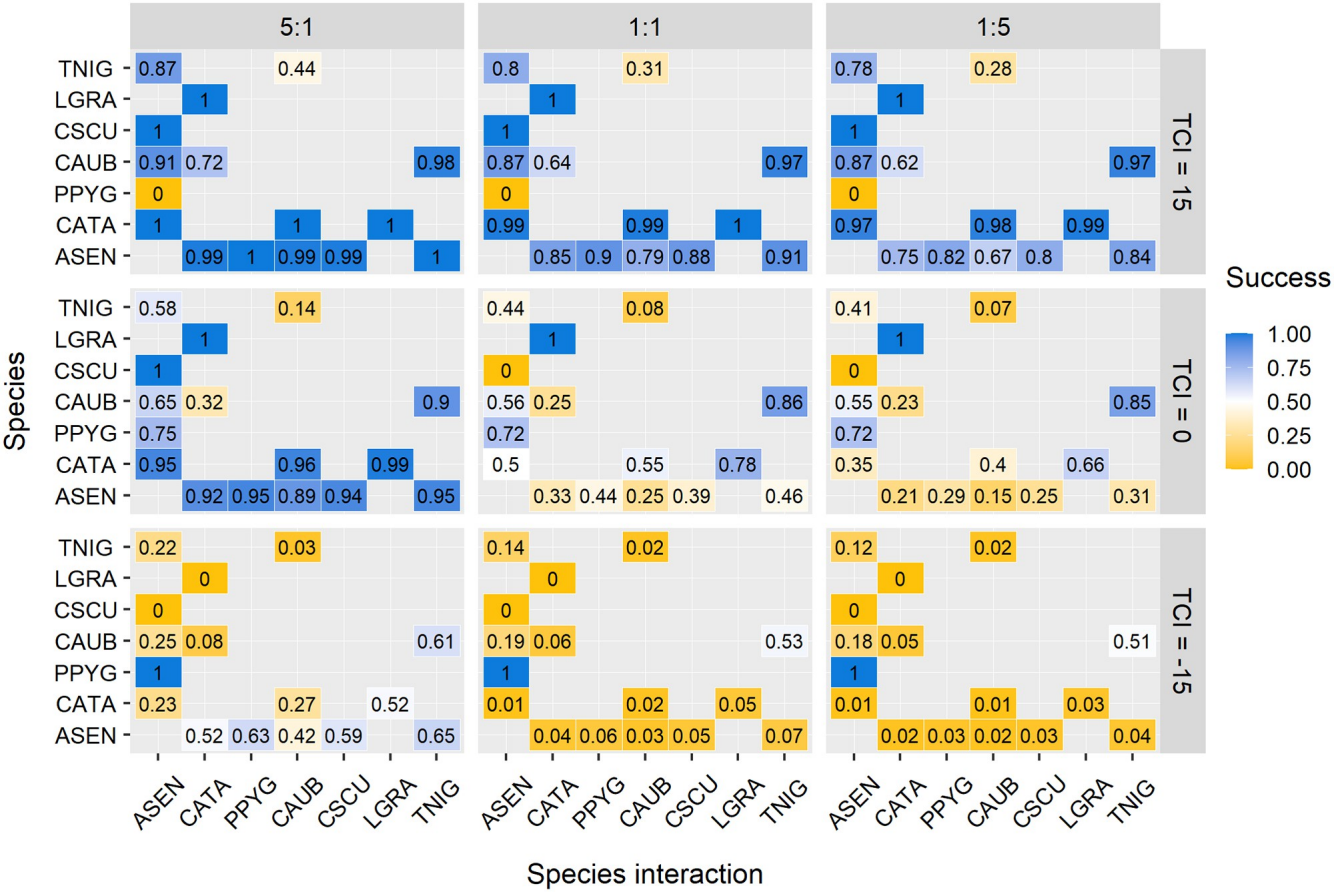
Pairwise comparisons showing the probability of success for the most common species-species interaction at three levels of worker proportion (5:1, 1:1, 1:5) and three levels of the thermal competition index (15, 0, -15). The values express the probability of success for the x axis species. Color gradient represents the probability of success in the competitive interaction: orange indicates lower probability, while blue indicates higher probability. Numbers in each box indicate the probability. Squares separate the species’ interactions into subordinate-subordinate (bottom-left), dominant-subordinate (top-left and bottom-right) and dominant-dominant (top-right). Species abbreviations: Subordinates: ASEN (*Aphaenogaster senilis*), CATA (*Cataglyphis floricola* and *Cataglyphis tartessica*) and PPYG (*Plagiolepis pygmaea*); Dominants: CAUB (*Crematogaster auberti*), CSCU (*Crematogaster scutellaris*), LGRA (*Lasius grandis*) and TNIG (*Tapinoma* cf. *nigerrimum*).

By combining three levels of the thermal competition index with three levels of worker proportion, we performed a total of 16 prediction probabilities of success replacement for each variable value combination and each species (Fig 6). Four predictions were obtained from subordinate-subordinate interactions, ten from dominant-subordinate or subordinate-dominant interactions and only two from dominant-dominant interactions (dominant and subordinate terms are following [42]).

The success probability of a given species varied in the same way for all the species interactions depending on the combination of the variable values chosen. The combination of higher values of TCI (TCI = 15) and worker proportion (5:1 ratio) produced success probabilities close to 1 for most of the species’ interactions. Furthermore, negative values of TCI and a disadvantageous number of ants (1:5 ratio) reduce the success probabilities, with obtained values close to 0 for most of the species. Just one species followed the opposite trend for the TCI (*P*. *pygmaea*, Fig 6). A neutral value of TCI (TCI = 0) and a 1:1 ant rate can be used to order the species in a dominance hierarchy according to their success probabilities. This dominance hierarchy would be the one acting when removing the effect of temperature or numerical dominance in the interaction (by setting TCI to 0 and ant rate to 1:1). In such a case, neither of the two parameters benefit either of the two species, so we obtain the dominance hierarchy for a neutral scenario.

## Discussion

We integrated diverse factors that determine diurnal food exploitation by ant assemblages of three different Mediterranean habitats. There were no defined patterns of co-occurrence and temporal niche overlap, although ants were less abundant in the hottest hours of the day. In this sense, foraging activity (bait occupation) was driven by temperature (positive relationship in thermophilic species and negative relationship in heat-intolerant species). Lastly, we found that higher values of TCI (a new thermal competition index linking temperature changes to the maximal activity temperature of two interacting species) and worker proportion between interacting species produced higher success probabilities for replacements at resources.

Our results show how species from pitfall and bait captures co-occurred randomly in the different studied plots (Fig 2). Similar results have been recorded in other studies [37, 43]. Only one pine forest plot from the 14 studied plots showed a spatial segregation pattern for both trapping methods, because most of the plot was occupied by *T*. cf. *nigerrimum*, a dominant species capable of monopolizing resources [44], creating different community structures. It is reported that *T*. cf. *nigerrimum* is able to monopolize most benign microhabitats under *Retama sphaerocarpa* shrubs [45]. Dominant ants predominate numerically and they can occupy large territories excluding other dominant species [46]. In fact, in some arboreal ant communities, it has been shown that the co-occurrence pattern was segregated when considering only dominant species, while it was random when considering all the species [43]. In addition, a segregated pattern can occur during the coolest hours of the day, but not during the warmer ones; these patterns respond to changes in the thermal environment [36]. However, species from the pine forest did not show a marked species turnover throughout the day. Heat-intolerant dominant ant activity rhythms can differ depending on canopy cover, which gives a constant pattern in the pine forest, with the ants benefiting from the spatial heterogeneity having their period of activity lengthened [14, 28].

Temporal niche overlap followed a random pattern except in a savin forest plot and a pine forest plot where we obtained a segregated pattern, but it is not possible to identify which species are responsible of this segregated pattern. Temporal segregated patterns have been obtained in other studies [36, 37]. The random patterns we observed can be explained by the diversity of ant communities, where there are species adapted to the whole range of temperatures, so we had no gaps without activity throughout the day time.

During competitive interactions, we found that the effect of temperature (higher values of TCI) and numeric supremacy (higher worker proportion) gave higher success probabilities. Most competitive interactions were between dominant-subordinate species, while dominant-dominant species interactions were less common. Abundant species, which tend to win in competitive interactions, could be considered dominant [37], but see [15]. The terminology “dominant” and “subordinate” does not mean that subordinates exploit suboptimal niches, but they respond to changing environmental conditions without losing efficiency [47]. Both terms are context-dependent, taking into account the temperature to define them. Until now, most studies have used temperature differences as a predictor to study ant species replacement [14, 37]. Temperature differences are a good predictor when the MAT of the two interacting species is similar, however, TCI is a better predictor when the MAT of the two interacting species differs. In our case, some specific value predictions did not fit the model because both species of the interaction presented a similar MAT. The fact that TCI takes into account the change of temperature combined with the MAT of the interacting species improves the predictions about species efficiency. A good knowledge of MAT and critical thermal limit values of ant species in each ant community is a necessary step for obtaining an accurate TCI, because it has been shown that ant diet and environmental breeding conditions could affect thermal resistance values, e.g., enhanced carbohydrate nutrition or developing at warmer temperatures enable higher thermal tolerance [29, 48, 49].

To summarize, a relatively small area of less than 7,000 ha, the Doñana Biological Reserve, presents a mosaic of different contrasting habitats with very different thermal conditions. Each habitat offers ant species a different availability of nesting sites and food resources. However, as most species are omnivorous and ground-dwellers, the most important differences among the habitats arise from the environmental thermal conditions. In our study, habitats with a higher vegetation cover provide less thermal niches than open habitats. In the open habitats, ants have more temporal windows with different thermal conditions (seasonally and daily) and extreme thermal niches are exploited by subordinates [30, 50]. Instead, within Brazilian forests, that are typically shaded and cooler than open habitats, ground temperature drove local foraging ant diversity, and at higher temperatures, or on hotter days, more forest ant species were active [51]. Some recent works both in tropical and boreal habitats highlight the importance of circadian activity differences in functional diversity and competitive relationships within ant communities [52, 53]. Moreover, in a North American deciduous forest ant community, evidence for any of the commonly suggested mechanisms of coexistence (dominance-thermal tolerance tradeoff, spatial segregation, temperature-based niche partitioning, among others) was not detected; with the exception of ant species partitioning foraging times, with dominant species foraging more intensely at night, while subdominants foraged during the day [54]. In our study, even though we have not detected an effect of the activity differences between species based on time (random pattern of temporal niche), probably because Mediterranean habitats are thermally more heterogeneous, the modularity analysis showed a strong thermal niche co-ocurrence based on their optimal foraging temperature.

To conclude, our results confirm that ant communities are regulated either by temperature or competition depending on the habitat structure [50, 55]. In more homogeneous habitats (with more vegetation cover), where there is low temperature variability (i.e. thermal niches), there is a higher abundance of dominant ants and competition is the main factor structuring the community. In habitats more patchy (with lower vegetation cover), where temperature fluctuation leads to a wider range of thermal niches, temperature is the main structuring factor, turning competition into a secondary factor of each specific thermal niche, and allowing the thermally-segregated coexistence of dominant and subordinate species.

## Supporting information

S1 Appendix ImR R code. (R)

S2 Appendix Regression stats. (XLSX)

S3 Appendix Datasets. (XLSX)

S1 Fig Bait occupation by temperature in each habitat. Dry scrubland (a), savin forest (b) and pine forest (c). Species abbreviations: ASEN (*Aphaenogaster senilis*), CAUB (*Crematogaster auberti*), CFLO (*Cataglyphis floricola*), CSCU (*Crematogaster scutellaris*), CTAR (*Cataglyphis tartessica*), LGRA (*Lasius grandis*), Miscelaneous (less abundant species), PPAL (*Pheidole pallidula*), PPYG (*Plagiolepis pygmaea*), SSP1 (*Solenopsis* sp.), TFOR (*Tetramorium forte*), TNIG (*Tapinoma* cf. *nigerrimum*), TSP1 (*Temnothorax* sp. 1) and TSP2 (*Temnothorax* sp. 2).
(TIF)

S1 Table List of species sampled in each habitat. (DOCX)
