# Supplementary figures and images for: Temperature or competition: Which has more influence on Mediterranean ant communities?
Source: PLoS One. 2022 Apr 29;17(4):e0267547. doi: 10.1371/journal.pone.0267547 (PMC9053807; doi:10.1371/journal.pone.0267547)

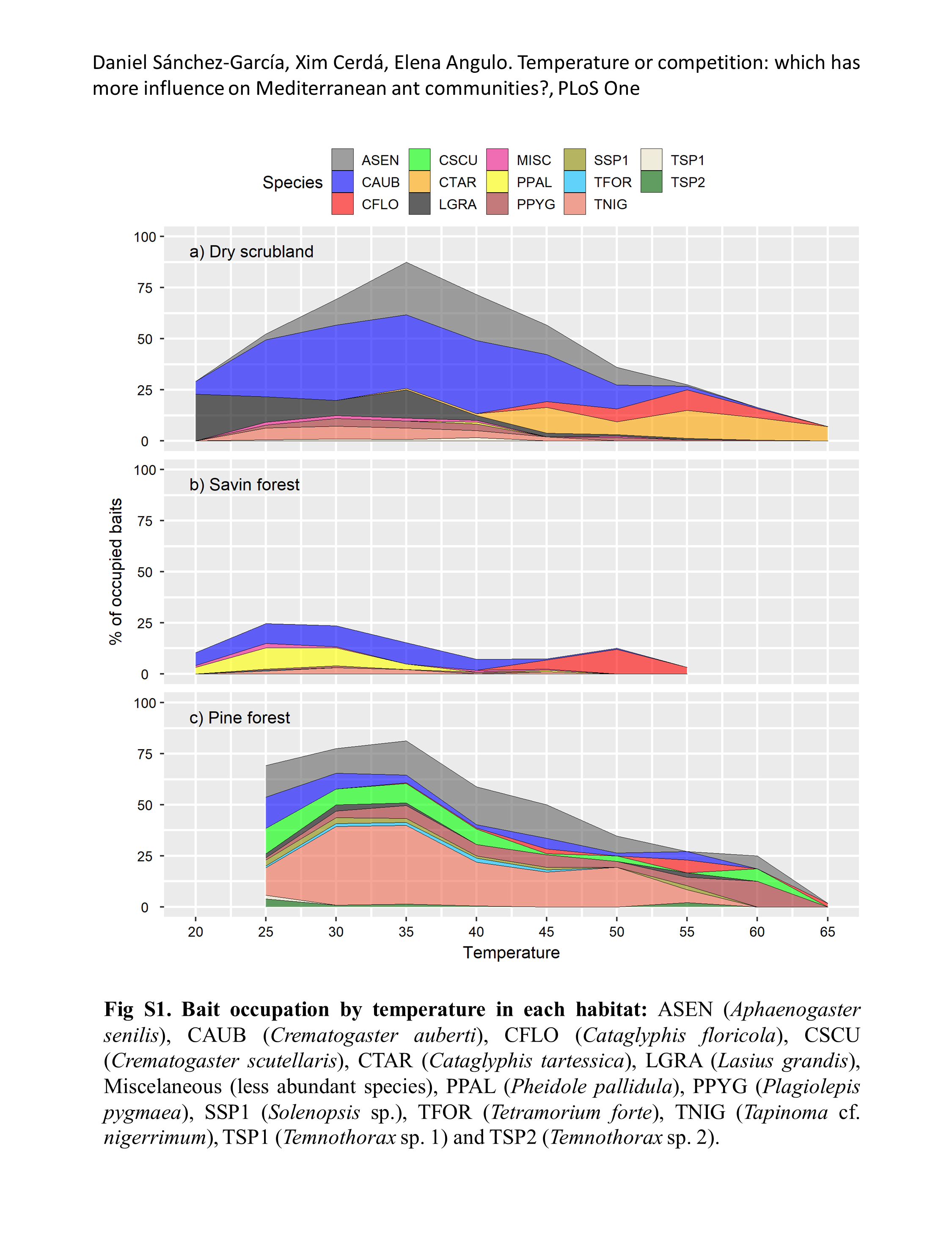

Supplement: S1 Fig — Dry scrubland (a), savin forest (b) and pine forest (c). Species abbreviations: ASEN (Aphaenogaster senilis), CAUB (Crematogaster auberti), CFLO (Cataglyphis floricola), CSCU (Crematogaster scutellaris), CTAR (Cataglyphis tartessica), LGRA (Lasius grandis), Miscelaneous (less abundant species), PPAL (Pheidole pallidula), PPYG (Plagiolepis pygmaea), SSP1 (Solenopsis sp.), TFOR (Tetramorium forte), TNIG (Tapinoma cf. nigerrimum), TSP1 (Temnothorax sp. 1) and TSP2 (Temnothorax sp. 2). (TIF) [file pone.0267547.s004.tif]
