# Supplementary material for: Temperature or competition: Which has more influence on Mediterranean ant communities?
Source: PLoS One. 2022 Apr 29;17(4):e0267547. doi: 10.1371/journal.pone.0267547 (PMC9053807; doi:10.1371/journal.pone.0267547)
Supplement: S1 Table — (DOCX) [file pone.0267547.s005.docx]

**Daniel Sánchez-García, Xim Cerdá, Elena Angulo. Temperature or competition: which has more influence on Mediterranean ant communities? – PLoS One**

**Table S1.** Species sampled in each habitat. We give the total number of ants of each species caught in all pitfall traps in each habitat. We sampled 2 plots in each habitat except for pine forest for which 4 plots were sampled (see Fig 1).

| ***Subfamily***/*Species* | Savin Juniper forest | Dry scrubland | Pine forest |
| --- | --- | --- | --- |
|  |  |  |  |
| ***Dolichoderinae*** |  |  |  |
| *Tapinoma nigerrimum* | 20 | 156 | 1145 |
| **Total** | **20** | **156** | **1145** |
|  |  |  |  |
| ***Formicinae*** |  |  |  |
| *Camponotus fallax* | 0 | 0 | 8 |
| *Camponotus lateralis* | 0 | 0 | 21 |
| *Camponotus pilicornis* | 0 | 16 | 15 |
| *Camponotus ruber* | 0 | 1 | 0 |
| *Cataglyphis floricola* | 13 | 125 | 5 |
| *Cataglyphis tartessica* | 0 | 491 | 6 |
| *Iberoformica subrufa* | 0 | 14 | 0 |
| *Lasius grandis* | 0 | 27 | 49 |
| *Plagiolepis schmitzii* | 3 | 5 | 146 |
| **Total** | **16** | **679** | **250** |
|  |  |  |  |
| ***Myrmicinae*** |  |  |  |
| *Aphaenogaster gibbosa* | 1 | 2 | 0 |
| *Aphaenogaster senilis* | 0 | 94 | 152 |
| *Crematogaster auberti* | 24 | 141 | 13 |
| *Crematogaster scutellaris* | 0 | 0 | 43 |
| *Messor marocanus* | 0 | 1 | 0 |
| *Myrmica aloba* | 1 | 0 | 0 |
| *Oxyopomyrmex saulcyi* | 2 | 0 | 0 |
| *Pheidole pallidula* | 87 | 0 | 0 |
| *Solenopsis*sp*.* | 0 | 0 | 2 |
| *Temnothorax*cf*. racovitzai* | 3 | 1 | 242 |
| *Temnothorax pardoi* | 0 | 0 | 11 |
| *Temnothorax tyndalei* | 30 | 45 | 36 |
| *Tetramorium caespitum* | 5 | 8 | 2 |
| **Total** | **153** | **292** | **501** |
|  |  |  |  |
| **TOTAL: 3212 ants** | 189 | 1127 | 1896 |
